# MicroRNA Regulation of Human Genes Essential for Influenza A (H7N9) Replication

**DOI:** 10.1371/journal.pone.0155104

**Published:** 2016-05-11

**Authors:** Stefan Wolf, Weilin Wu, Cheryl Jones, Olivia Perwitasari, Suresh Mahalingam, Ralph A. Tripp

**Affiliations:** 1 Department of Infectious Diseases, University of Georgia, Athens, GA, United States of America; 2 Institute for Glycomics, Griffith University, Gold Coast, Southport, QLD, Australia; Centre of Influenza Research, The University of Hong Kong, HONG KONG

## Abstract

Influenza A viruses are important pathogens of humans and animals. While seasonal influenza viruses infect humans every year, occasionally animal-origin viruses emerge to cause pandemics with significantly higher morbidity and mortality rates. In March 2013, the public health authorities of China reported three cases of laboratory confirmed human infection with avian influenza A (H7N9) virus, and subsequently there have been many cases reported across South East Asia and recently in North America. Most patients experience severe respiratory illness, and morbidity with mortality rates near 40%. No vaccine is currently available and the use of antivirals is complicated due the frequent emergence of drug resistant strains. Thus, there is an imminent need to identify new drug targets for therapeutic intervention. In the current study, a high-throughput screening (HTS) assay was performed using microRNA (miRNA) inhibitors to identify new host miRNA targets that reduce influenza H7N9 replication in human respiratory (A549) cells. Validation studies lead to a top hit, hsa-miR-664a-3p, that had potent antiviral effects in reducing H7N9 replication (TCID_50_ titers) by two logs. *In silico* pathway analysis revealed that this microRNA targeted the LIF and NEK7 genes with effects on pro-inflammatory factors. In follow up studies using siRNAs, anti-viral properties were shown for LIF. Furthermore, inhibition of hsa-miR-664a-3p also reduced virus replication of pandemic influenza A strains H1N1 and H3N2.

## Introduction

Influenza virus is still a serious global health threat affecting humans, wildlife and agricultural species. Human infection with avian influenza A H7N9 virus (H7N9) were first reported in China in March 2013 [1] Most of the infections are believed to have resulted from exposure to infected poultry or contaminated environments, as H7N9 viruses have been found in poultry in China. While some mild illnesses in humans infected with H7N9 has been reported, most patients experienced severe respiratory illness, such as pneumonia (97.3%) and acute respiratory distress syndrome (71.2%), leading to high rates of intensive care unit admissions [2]. Human mortality attributed to influenza H7N9 is over 38% with 175 deaths from 450 confirmed cases within a 20-month period [3]. No evidence of sustained human-to-human transmission of H7N9 has been recorded; however, there was some evidence for limited person-to person spread under rare circumstances [4]. H7N9 began in China, but now has rapidly spread to other countries [5]. Recently, the first documented case of H7N9 in humans was reported for North America in Canada [6]

No vaccine is currently available for H7N9 [7]. There are several drugs available for the treatment of influenza infections including the M2 ion channel inhibitors amantadine and rimantadine, and the neuraminidase inhibitors, zanamivir and oseltamivir [8, 9]. Early treatment with these antiviral drugs has been shown to reduce the duration of symptoms and time to recovery, however, the use of antiviral drugs is complicated by the emergence of drug resistant viruses [10, 11]. Consequently, oseltamivir-resistant H7N9 strains have already been described in recent reports from Taiwan [12]. In addition, the use of antiviral drugs may have an effect on population vulnerability due to lack of seroconversion, as well as driving drug resistance among circulating strains [13]. To prevent the spread of infection, new drug and vaccine development is needed. However, difficulties include a lack of understanding of the host factors required for replication, and unusual mutations that occur in the virus that differ from other avian influenza viruses [14].

Linking high-throughput screening (HTS) with RNA interference (RNAi) allows for the rapid discovery of the molecular basis of disease pathogenesis, and the identification of potential pathways for the development of safe and effective treatments. Recent advances in our understanding of RNAi have allowed for genome-wide screens to determine and validate the host cell genes that may are required for influenza virus replication [15] Small interfering RNA (siRNA) can be readily developed to target viral or host genes, and have been successfully applied in disease intervention approaches. For example, siRNA targeting respiratory syncytial virus has been efficacious for silencing virus replication [16]. In the case of influenza, inhibiting the host gene CAMK2B prevented virus replication *in vitro* [17], and knocking down trypsin also inhibited virus replication and apoptosis [18]. In a siRNA screen of 481 human protease genes in A549 cells, 5 genes, ADAMTS7, CPE, DPP3, MST1 and PRSS12, were identified as essential for influenza virus replication [19]. Another siRNA screen of 720 human protein kinase genes (HPK), 17 HPKs were validated as essential for influenza A replication [20]. In both screens vital genes for influenza A replication were found that affect multiple host pathways and that are regulated by microRNAs (miRNAs) induced during infection.

**Fig 1:**
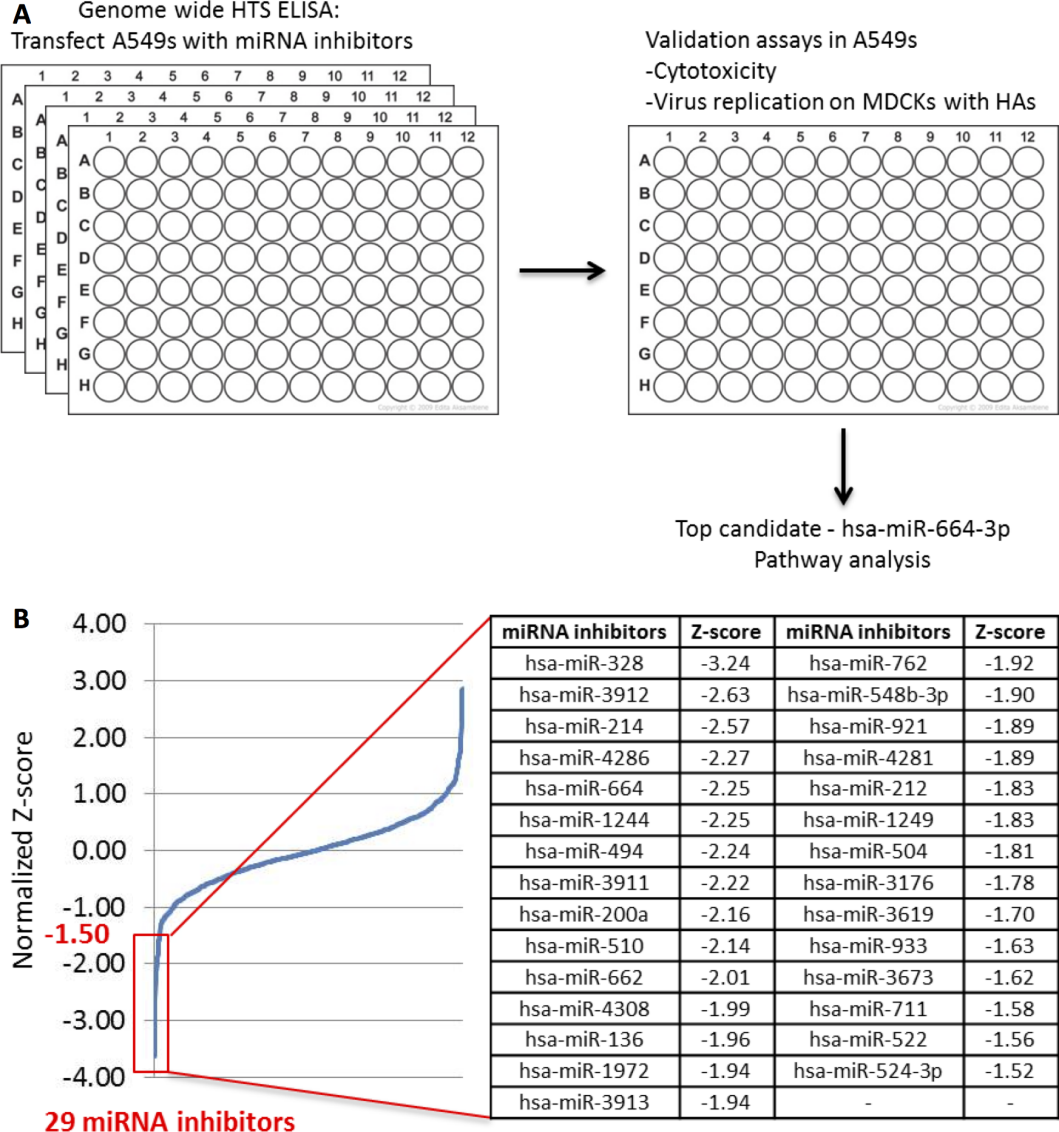
Workflow from HTS to pathway analysis of miRNA top hits. (A) A549 cells were reverse-transfected with a library of 1,200 miRNA mimics at a final concentration of 20 nM. Non-targeting miRNA mimic controls and siRNAs (siNP) to countermeasure H7N9 replication were used as negative and positive controls, respectively. 48 h after miRNA transfection, the cells were infected with H7N9 at MOI of 0.01 for 24 h. 24 h after infection, the plates were fixed and analyzed by ELISA. 10 top hits from HTS ELISA were validated in virus replication studies. Top hit has-miR-664a-3p was then investigated for pathway analysis and downstream target effects. (B) A plot of normalized Z-score values calculated based on the ELISA results showing a wide range of virus replication modulation. All miRNAs with a Z-score < -1.5 were subjected to further validation.

In addition to host gene involvement during viral infection, the magnitude and tempo of host gene expression is governed by factors such as miRNA. miRNAs have been used to validate the impact of host genes on virus replication and have been used as therapeutics [21] with the ability to negatively affect influenza replication [22]. Thus, host miRNAs have a role in host gene expression in response to influenza infection. miRNAs are small noncoding single-stranded RNA molecules composed of 18–23 nucleotides which regulate gene expression in eukaryotes. The miRNA family is a global regulatory network controlling homeostasis, inflammatory responses affecting immunity and disease pathogenesis [23]. miRNAs are involved in the degradation of cytokine transcripts and modulate the expression of negative regulators of cytokine expression and signaling pathways [24]. Paired analysis of miRNA inhibitors and mimics enables gain and loss of function studies for a given miRNA. miRNA can not only be used as a tool for screening, but also as a therapeutic itself. The drug miravirsen is an anti-miRNA drug candidate currently in late stage clinical evaluation for treatment of hepatitis C virus (HCV) infections. Miravirsen is thought to work mainly by hybridizing to mature miR-122 and blocking its interaction with HCV RNA [25]. In this study, a genome wide HTS was performed to identify target miRNAs as countermeasures of H7N9 replication. Several pro-viral host miRNA’s were identified which are important for H7N9 replication. In validation studies, the most promising target, hsa-miR-664a-3p (miR-664), was further investigated and the effects on downstream genes, Leukemia inhibitory factor (LIF) and NIMA-related kinase 7 (NEK7), were explored (Fig 1A).

## Results

### Genome wide high-throughput screen of miRNAs that govern influenza H7N9 replication

A human genome wide HTS was performed in A549 cells using miRNA inhibitors to reveal possible host targets that are important for H7N9 replication. Z-score (the number of standard deviations from the mean) was used to normalize data to provide explicit information on the strength of each miRNA inhibitor relative to the rest of the sample distribution. A negative z-score reveals inhibitors that decrease virus replication, where a positive z-score reflects miRNA inhibitors that increase virus replication. All miRNAs inhibitors having a z-score lower than -1.50, 29 in total, were investigated (Fig 1B). Cytotoxicity assays were performed to rule out miRNA inhibitors with a toxic effect on cells (data not shown). Finally, 10 miRNA top hits with the most negative z-scores were identified (Table 1) for validation studies.

**Table 1:** List of miRNA top hits from HTS with z-scores.

miRNA inhibitors Z-scores*			
miR-136	-3.62	miR-664	-2.25
miR-328	-3.24	miR-1244	-2.25
miR-3912	-2.63	miR-3911	-2.22
miR-380	-2.62	miR-510	-2.14
miR-4286	-2.27	miR-662	-2.01

* Z-score: the number of standard deviations from the mean. A negative z-score represents the pro-viral character of the corresponding miRNA. A more negative z-score reflects a greater importance of the miRNA for virus.

### Validation of top 10 miRNA inhibitors that reduce influenza A H7N9 replication

**Fig 2:**
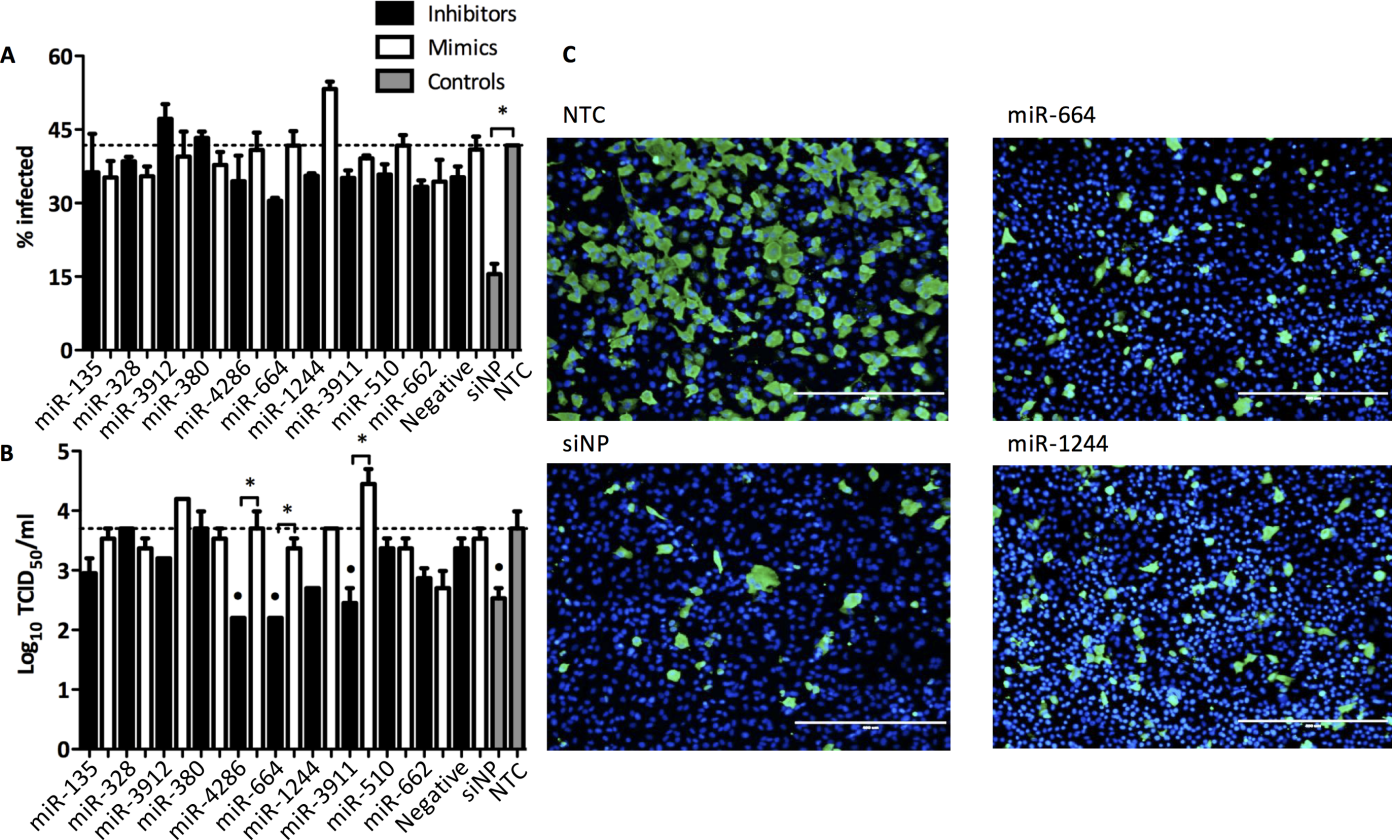
Validation of miRNA inhibitor top hits that reduce H7N9 replication. (A-B) A549 were transfected with miRNA inhibitors and mimics at 25 nM for 48 h and then infected with H7N9 at MOI 0.01 for 48 h. Cells were fixed with 10% formalin and stained for viral NP and counterstained with DAPI. Stained cells were visualized and analyzed using Cellomics ArrayScan high content imaging system. NTC; Non-transfected control, siNP; Positive control (A) Percentage of infected cells was graphed. The data are from 3 replicate wells +/- SEM. *p<0.05. (B) Supernatants from transfected and infected A549 cells were titrated on MDCK cells. The data are from 3 replicate wells ± SEM. *p<0.05, °p<0.05 compared to positive. (C) Representative fluorescent microscope images were taken with EVOS FL imaging system (Life Technologies). Scale bar, 400 μm. NP, green; DAPI (nuclei), blue.

To validate the top 10 miRNAs from the HTS, A549 cells were transfected (25 nM) with miRNA inhibitors or mimics, and then infected (MOI 0.01) with H7N9. Supernatants and cell monolayers were processed to investigate virus replication. Cellomics ArrayScan (ThermoScientific) was used to count the number of infected cells by using fluorescent viral nucleoprotein (NP)-staining combined with DAPI (4',6-diamidino-2-phenylindole) staining of the nuclei. Approximately 40% of cells were infected in the non-transfected control (NTC) (Fig 2A). Reduction of H7N9 infectivity, as predicted from HTS, was confirmed in validation studies for 8 miRNAs. Inhibition of H7N9 replication by miRNA-664 was confirmed where infectivity of H7N9 was reduced by 27% (Fig 2B). siNP, which targets the viral nucleoprotein (NP), was used as positve control for the inhibition of H7N9 replication. siNP reduced replication of H7N9 by 63%. Negative controls having minimal sequence identity were used for all miRNA inhibitors and mimics to differentiate between specific and non-specific effects, and the negative transfection controls had no impact on virus replication.

The pro-viral properties of miR-664 were confirmed as the inhibitor of miR-664, miR664i, significantly (p<0.05) reduced virus replication by nearly 2 logs. siNP, as the positive control, significantly (p<0.05) reduced H7N9 replication by 1 log. Negative transfection controls had no impact on virus replication. Together, miR664i revealed the most potent antagonizing effect on H7N9 replication and was therefore selected to investigate downstream events and to perform host pathway analysis. Other potent miRNAs regulating H7N9 replication, e.g. hsa-miR-1244 and hsa-miR-4286, were not further investigated in this study, but is the subject of a burgeoning study.

### Identification of miRNA gene targets during influenza A H7N9 infection

**Fig 3:**
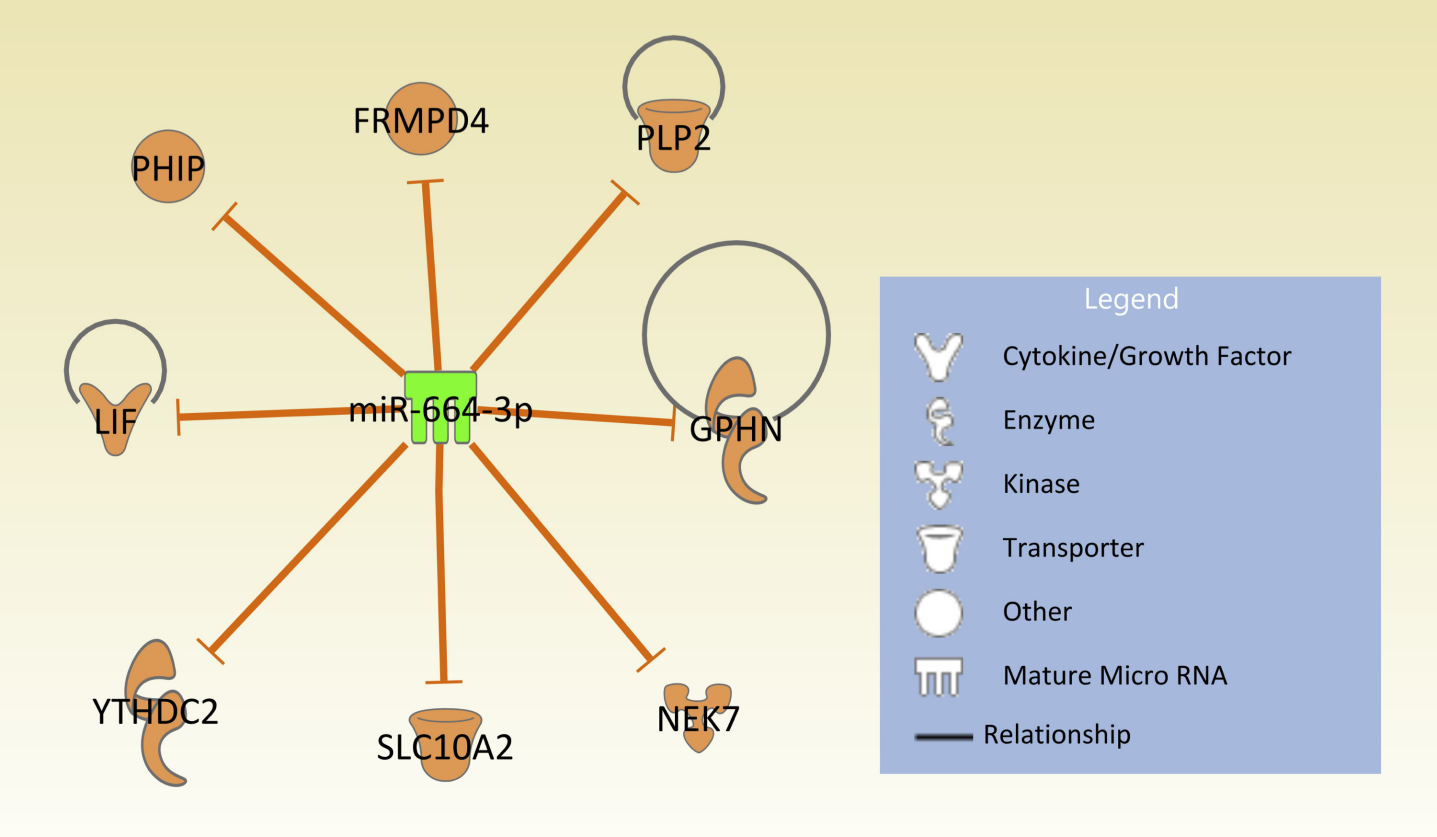
Eight highly predicted target genes of miR-664. Ingenuity pathway analysis was used to determine target genes of miR-664. Only predicted molecules with high confidence are displayed. Molecules are part of different proteins such as cytokines, enzymes, kinases, transporters or as other if the function is unknown. Orange arrows show inhibitory effects of miR-664 on target genes. Pathway diagram was created using Qiagen IPA Path Designer.

To determine whether miR-664 inhibitor, miR664i, targets influenza A (H7N9) or impairs virus replication by affecting the host cell machinery, a BLAST (Basic Local Alignment Search Tool) search was performed to compare miR-664 and H7N9 virus microRNA seed sites. No noteworthy microRNA seed sites in the H7N9 sequence were found (fludb.org), so it is unlikely the miRNA inhibitor is directly targeting viral gene expression. Thus, miR-664 may be a pro-viral miRNA, required by the virus to downregulate downstream target genes which are important for viral defense mechanisms by the host cell. Pathway analysis using Ingenuity Pathway Analysis (IPA) was used to explore downstream targets of miR-664. A data set was created from the targetscan.org database using the miRNA target scan option in IPA. Since there was no experimental data available for miR-664, results were filtered for targets with highly-predicted confidence for an interaction with miR-664. The IPA target scan produced a pathway for 8 highly predicted targets of miR-664 and their predictive interactions (Fig 3).

Pathways of the predicted target genes for miR-664 are not well characterized; however, some are known to have ubiquitous functions in the cell (Table 2). Specific focus was set on molecules LIF and NEK7. LIF and NEK7 may be linked to regulatory functions in cytokine pathways such as the pro-inflammatory cytokines interleukin-6 (IL-6) and IL-12a and the chemokine CXCL10 (Table 3). Reports from H7N9-infected humans have shown significant upregulation of IL-6 and CXCL10 compared to healthy controls [2, 26–29]. In a study on mice, IL-6 was shown to be a main driver of lung pathology [30]. An interesting finding was that the expression of IL-12a was upregulated with other avian influenza viruses, but not in cells infected with H7N9 [31]. Therefore, roles of LIF and NEK7 in the pro-viral effect of miR-664 were further investigated.

**Table 2:** Highly predicted target molecules/genes for miR-664 by IPA software.

Symbol	Pathway
FRMPD4	Unknown
GPHN	GABA Receptor Signaling, Molybdenum Cofactor Biosynthesis
LIF	ERK5 Signaling, Hematopoiesis from Pluripotent Stem Cells, Hepatic Cholestasis, HMGB1 Signaling, Mouse Embryonic Stem Cell Pluripotency, Role of NANOG in Mammalian Embryonic Stem Cell Pluripotency, Role of NFAT in Cardiac Hypertrophy, Role of Pattern Recognition Receptors in Recognition of Bacteria and Viruses
NEK7	Unknown
PHIP	Unknown
PLP2	Unknown
SLC10A2	FXR/RXR Activation, Hepatic Cholestasis
YTHDC2	Unknown

**Table 3:** Regulatory relationship of LIF and NEK7.

Molecule	Family	Regulates	Regulated by
LIF	Cytokine	STAT3, SOCS3, POMC, Erk1/2, IL6ST, STAT1, LIFR, SOCS1, IL-6*, FGF5, FOS, Alp, T, KDR, POU5F1	lipopolysaccharide, TNF, IL-1β, progesterone, TGFB1, IL-2, Il-1, dexamethasone, LPP, MAPK8, Lethal toxin, TP53, AGT, phorbol myristate acetate, Tgf beta
NEK7	Kinase	EDN1, RGCC, LMO4, IL-12A*, U90926, ACPP, ISG15, PTGS2, CXCL10*	TLR4, PLK, triamcinolone acetonide

* pro-inflammatory cytokines and chemokines

### Regulation of miR-664 expression and host gene targets during Influenza H7N9 infection

**Fig 4:**
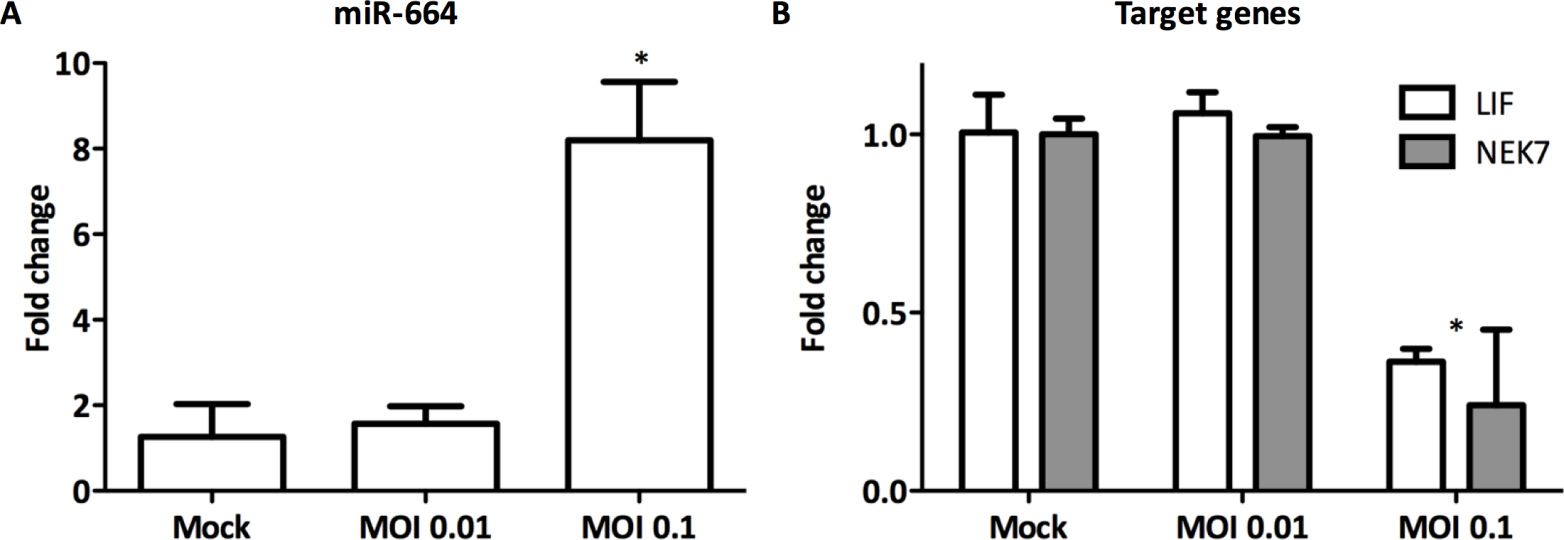
Effect of influenza A (H7N9) on gene expression levels of miR-664 and target genes. A549 cells were infected with H7N9 at MOI 0.01 and 0.1 for 24 h. RNA was extracted for gene expression analysis of A) LIF and NEK7, and B) miR-664 using RT-qPCR. Expression is normalized to 18S and relative to non-infected cells. Data are from 3 replicate wells ± SEM. *p<0.05.

RT-qPCR was used to investigate the gene expression levels of miR-664 and predicted host target genes, LIF and NEK7, during infection with H7N9. miR-664 was upregulated nearly 10-fold (p<0.05) in A549 cells infected (MOI = 0.1) with H7N9 at 24 h pi (hours post-infection) compared to non-infected A549 cells (Fig 4). Importantly, potential target genes LIF (p<0.01) and NEK7 (p<0.05) were significantly downregulated, indicating a correlation between miR-664 and the target genes predicted by IPA.

**Fig 5:**
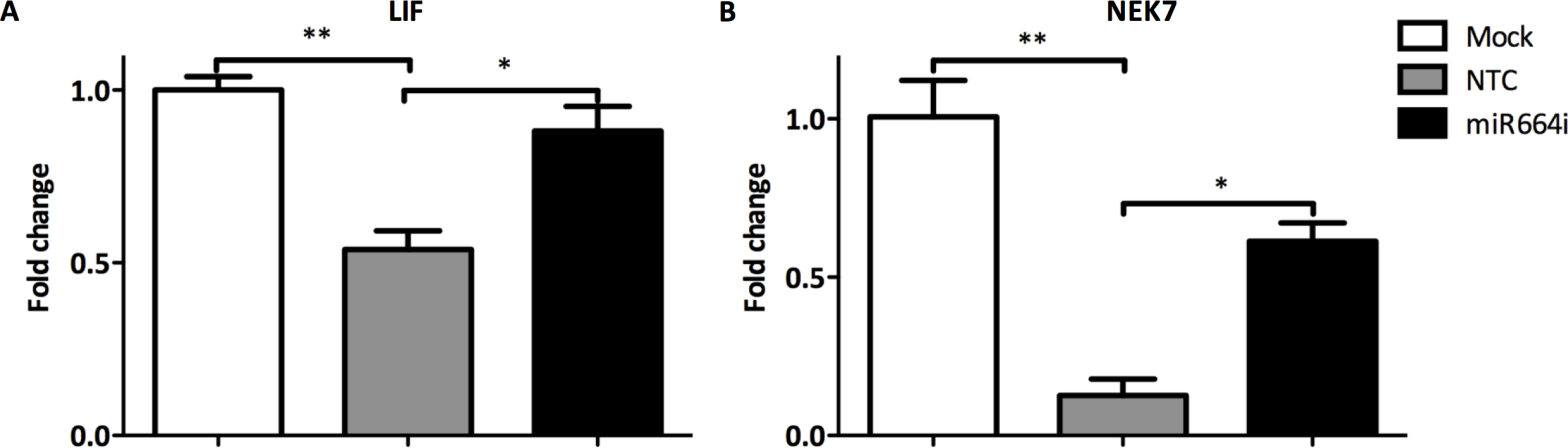
Impact of miR664i on expression of target genes during influenza H7N9 infection. A549 were transfected with miR664i and then infected with H7N9 at MOI = 0.1 for 24 h. Gene expression of target genes LIF and NEK7 were measured with qRT-PCR to evaluate the impact of miR664i on target molecule expression during H7N9 infection. Gene expression was normalized to 18S and relative to non-infected cells. (3 replicate wells) ± SEM. *p<0.05, **p<0.01.

Follow up studies aimed to investigate whether there was a direct effect of miR-664 inhibitor, miR664i, on the gene expression levels of the target genes during infection with H7N9. A549 cells were transfected with miR664i prior to infection with H7N9. LIF and NEK7 gene expression was downregulated during H7N9 infection compared to non-infected control cells as previously observed (Figs 4 and 5). LIF and NEK7 gene expression levels were rescued when the cells had been transfected with miR664i prior to infection (Fig 5). Thus, miR-664 may have a direct impact on target genes LIF and NE7 and their expression levels during influenza A H7N9 infection.

### Target gene knock-down identifies antiviral properties

**Fig 6:**
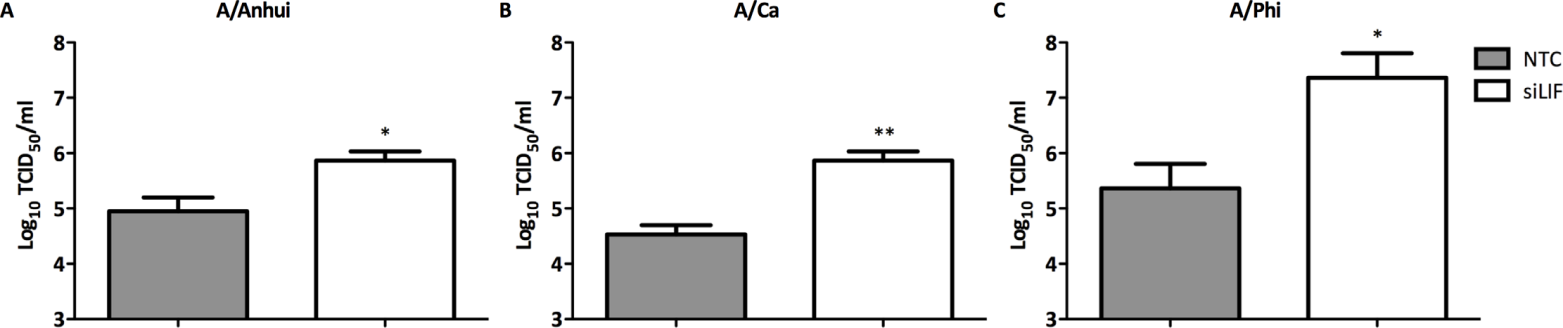
Knock-down of LIF enhances the replication of influenza A. A549 cells were transfected with siRNA against LIF 50 nM for 48 h. Cells were infected with A) A/Ca (H1N1) at MOI 0.1, B) A/Phi (H3N2) at MOI 0.05 and A/Anhui (H7N9) at MOI 0.01. At 48 h pi, supernatants were collected for virus titration in MDCK cells. (3 replicate wells) ± SEM. *p<0.05, **p<0.01.

Small interfering RNAs targeting LIF (siLIF) or NEK7 (siNEK7) were used to investigate the role of target genes LIF and NEK7 on virus replication (Fig 6). A549 cells were transfected (50 mM) with siRNA against LIF and NEK for 24 h and then the A549 cells were infected (MOI = 0.1) with A/Anhui (H7N9), A/Ca (H1N1) and A/Phi (H3N2) to determine the role of these genes in influenza A virus replication. Knock-down of LIF increased virus titers for all three influenza A strains investigated, indicating an important role of LIF in virus defense. Knocking down NEK7 did not impair virus replication, and thus this target gene may not be a critical factor in defense against influenza A viruses (data not shown). All siRNAs had prior been evaluated for their efficacy in downregulating the specific mRNA by RT-qPCR (S1 Fig). These results demonstrate that combining HTS and RNAi can lead to the rapid discovery of host cellular targets and the underlying pathways that participate in virus replication.

### miR664i reduces the replication of pandemic influenza A strains A/Ca and A/Phi

**Fig 7:**
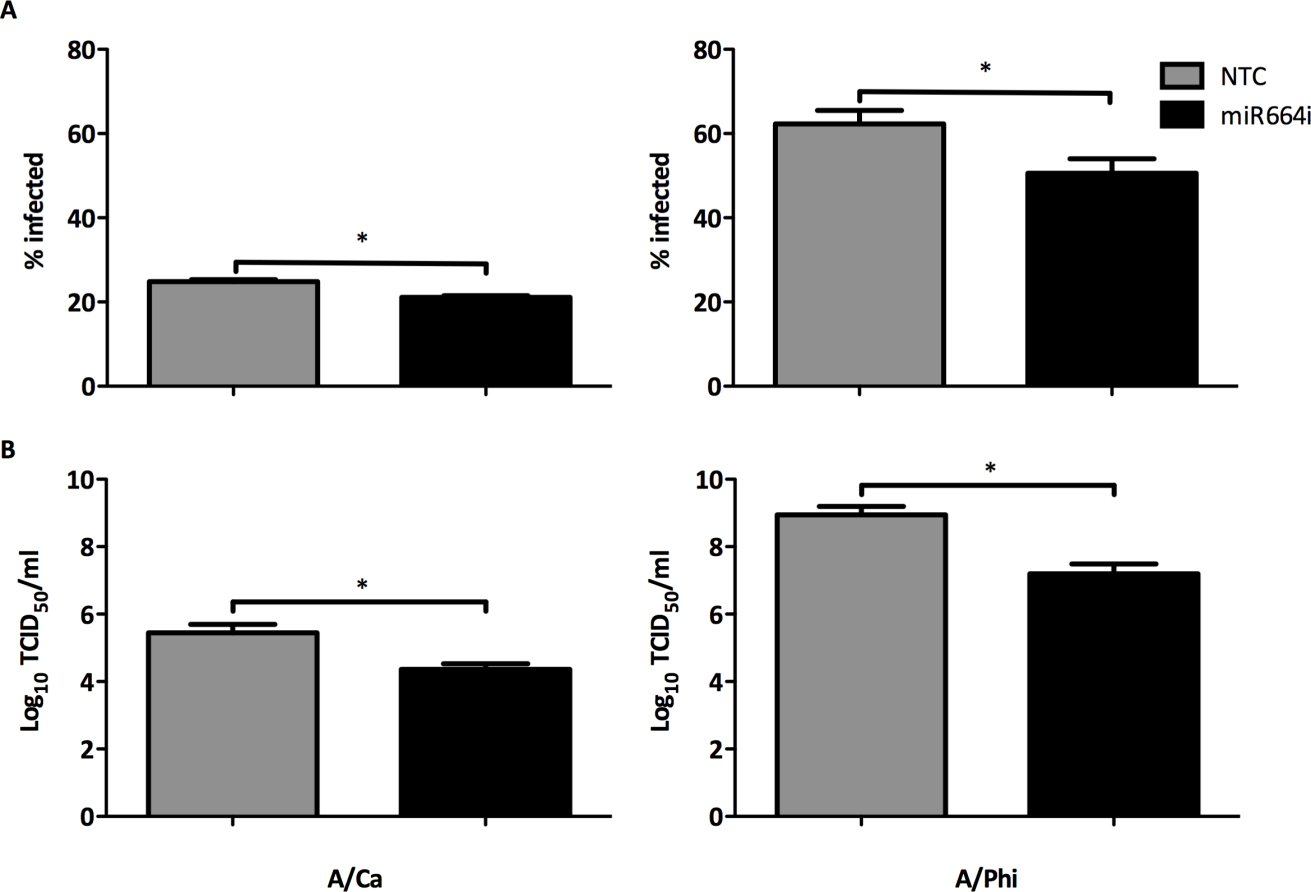
miR664i reduces the replication of other influenza A strains. (A-B) A549 cells were transfected with miR664i at 25 nM for 48 h. Cells were infected with A/Ca (H1N1) at MOI 0.1 and A/Phi at MOI 0.05 for 48 h. NTC; Non-transfected control (A) Cells were fixed with 10% formalin and stained for viral NP and counterstained with DAPI. Percentage of infected cells was measured with Cellomics ArrayScan and graphed. Data are from 3 replicate wells +/- SEM. *p<0.05. (B) Supernatants from transfected and infected A549 cells were titrated on MDCK cell. TCID_50_ values were calculated and graphed. (3 replicate wells) ± SEM.　 *p<0.05.

In order to investigate potential broad-spectrum antiviral effects of miR664i, A549 cells were transfected with miR664i at 25 nM for 48 h and subsequently infected with with A/Ca (H1N1) at MOI 0.1 and A/Phi at MOI 0.05 for 48 h. miR664i reduced both cell infectivity and virus titers for both virus strains investigated (Fig 7) by almost 2 logs TCID_50_. Targeting the host cell machinery to countermeasure influenza A H7N9 helped to discover a miRNA with broad pro-viral characteristics. Inhibition of miR664 limited the replication of various influenza A strains, including H7N9 and the pandemic H1N1 and H3N2, without putting evolutionary pressure on the virus which could potentially lead to resistant mutations.

## Discussion

To our knowledge this is the first report on a miRNA-based antiviral therapeutic that countermeasures H7N9 replication *in vitro*. miR664i, an inhibitor of host cell miRNA miR-664, showed potent antiviral effects inhibiting H7N9 replication by two logs (TCID_50_ titers) in A549 cells. A549 cells are a good proxy to establish and characterize an *in vitro* model of Type II respiratory alveolar epithelium, and as α2,3-linked or α2,6-linked sialic acids in principle dictate influenza virus susceptibility, A549 cells are highly susceptible to influenza infection and have been widely used as a cell culture model to study influenza A for almost two decades [32]. We anticipate examining the effects of miR664i on influenza A replication in BEAS2B cells or differentiated NHBE cells. In addition, we aim to investigate the antiviral potential of miR664i in animal models. There have been numerous successful attempts to increase potency and stability and to reduce off-target effects of RNAi therapeutics in recent years [33]. Locked-nucleid acid (LNA) modification can improve the serum stability, where the design of short hairpin RNA (shRNA) can be used as potent RNAi triggers. Lipid nanoparticles as carriers and adenovirus-mediated RNAi against viral infections have also successfully been applied [34, 35]. *In vivo* studies showed a reduction of hepatitis B virus (HBV) when mice were injected with LNA-modified RNAi therapeutics [36]. The most prominent use of LNA technology is the clinical candidate miravirsen, where monthly subcutaneously application can downregulate miR-122 and subsequently reduce HCV replication [37, 38]. In future studies, we will implement some of these techniques to investigate the potential of miR664i to countermeasure influenza A replication in a mouse model.

In a recent publication, MIR2911, a broad spectrum antiviral miRNA suppressed replication of H1N1, H5N1 and H7N9 influenza virus in *vitro* and *in vivo* [39]. MIR2911 targeted the PB2 and NS1 gene of influenza A, and considerably inhibited their protein expression. However, MIR2911 was ineffective against mutant influenza viruses in which the MIR2911-binding nucleotide sequences were altered. In contrast, miR664i does not target the virus directly and the antiviral effects may be directed through intracellular mechanisms in the host cell. One advantage of targeting the host cell miRNA, instead of the virus directly is the lower probability of the virus evolving mutations that would potentially lead to resistance against the therapeutic. In these studies, which examined mechanisms underlying the antiviral actions of miR664i, upregulation of miR-664 was observed during H7N9 infection of A549 cells. This is in accordance with a previous study investigating the expression of miRNAs in blood serum samples from H7N9-infected patients [40]. There, miR-664 expression was shown to be highly upregulated compared to healthy controls. Further, in a microarray screen of human blood serum from pandemic H1N1-infected patients and of H1N1-infected cell lines, miR-664 has been shown to be upregulated during infection [41]. However, whether the virus directly controls the upregulation of miR-664 in the cell, or if the virus takes advantage of the cells unbeneficial upregulation of miR-664 remains unclear.

**Fig 8:**
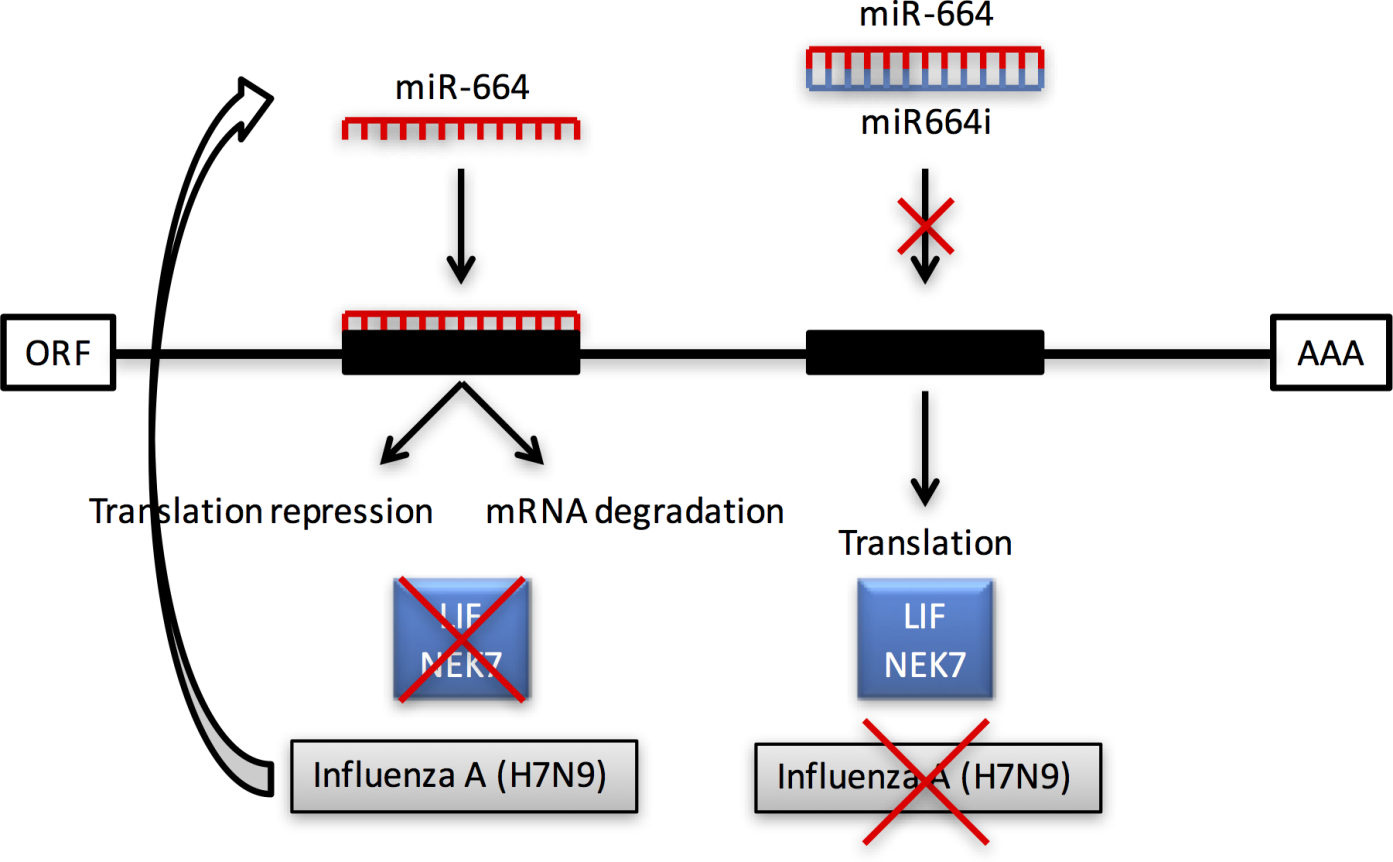
A model for miR-664-mediated regulation of influenza A H7N9 virus infection. miR-664 is upregulated during the course of influenza A infection of A549 cells, which inhibits the expression of target genes of LIF and NEK7. When an inhibitor of miR-664 is used, such as miR664i, molecules LIF and NEK7 are expressed normally, which counteracts the replication of influenza A.

The two molecules LIF (leukemia inhibitors factor), and NEK7 (NIMA-related kinase 7), were highly predicted target genes for miR-664based on the targetscan.org database. Gene expression studies showed that while miR-664 was upregulated during H7N9 infection, target genes LIF and NEK7 were downregulated. When miR664i was transfected prior to infection, expression levels of target genes were subsequently normalized. Thus, the inhibitory effect of miR-664 on its target genes during influenza infection may facilitate virus replication (Fig 8). A similar effect has been shown for miR-466I, which inhibits the host antiviral innate immune response against Sendai virus by targeting interferon (IFN)-α expression *in vitro* [42]. Unlike miR-466, no seed sites for miR-664 can be found in any of the human IFN genes. Therefore, a direct action of miR-664 IFN gene expression is not expected. However, it is possible that miR-664 can regulate virus-induced IFN production and secretion indirectly through other molecules in the IFN induction pathway. Furthermore, ongoing studies also aimed to address whether IFN regulates miR-664 expression in infected and non-infected cells. As these examples suggest, upregulation of specific miRNAs during virus infections can be harmful for the host. Nevertheless, several reports have shown that downregulation of other miRNAs can also result in increased virus replication.

In studies examining the furin-dependent proteolytic activation of highly pathogenic influenza H5 and H7 viruses, the miR-24 response was shown to strongly decrease both furin messenger RNA (mRNA) levels and intracellular furin activity in A549 cells [43]. Cells transfected with miR-24 mimics showed a decrease of H5N1 infectious virions and a complete block of H5N1 virus spread that was not observed in cells infected with H1N1 virus. This suggests that a virus-specific downregulation of furin-directed miRNAs such as miR-24 may represent a viral regulatory mechanism to govern the production of infectious virions.

To investigate the underlying mechanisms of miR-664 during influenza replication, knock-down studies using siRNA targeting LIF and NEK7 were performed. Knocking down NEK7 had no impact on virus replication, which suggests a non-relevant role of this particular kinase during influenza infection However, knocking down LIF increased virus replication in various influenza A strains. This suggests that miR-664 may downregulate LIF, which may be an important factor in immune defense against influenza A. Whether there are other factors involved and what happens downstream of LIF has yet to be determined. The importance of LIF in antiviral defense has been reported in previous publications [44]. *In vivo* studies were performed with LIF knock-out mice that were infected with RSV. LIF knock-out mice yielded higher virus titers compared to control mice. Furthermore, mice treated with anti-LIF IgG developed enhanced RSV pathology observed with increased airspace protein content, apoptosis and airway hyperresponsiveness compared to control IgG treatment. Thus, LIF may be a critical factor in the antiviral defense against RSV and other respiratory diseases such as influenza A. Whether this is solely mediated by miR-664, or by other mechanisms, remains unknown and will need to be investigated. Furthermore, it remains unclear if virus infection and /or replication initiates the upregulation of miR-664 to avoid the antiviral effects of LIF, or if this mechanism is a non-beneficial reaction by the host cell during virus infection.

In conclusion, the current study reports the discovery of an important pro-viral miRNA that can be targeted by readily available miRNA inhibitor miR664i. The field of miRNA inhibitors is on the rise with many candidates currently being tested in clinical trials. The most clinically advanced example of a miRNA antagonist is the antiviral miravirsen which is currently undergoing multiple clinical phase II studies against Hepatitis C (ClinicalTrials.gov identifier: NCT01200420, NCT02031133, NCT01872936, NCT01727934). Miravirsen targets the conserved host factor miR-122, which stimulates translation of HCV RNA. Thus, miR-122 is a promising target, as it is hoped that the virus would not be able to acquire resistance mutations to miravirsen. The promising antiviral candidate miR664i owns unique characteristics in the novel field of miRNA inhibitors as countermeasures against the potential pandemic and life-threatening infectious disease caused by H7N9. In addition, miR664i also shows great potential in treating pandemic circulating influenza A strains such as H1N1 and H3N2.

## Materials and Methods

### Cell lines and viruses

All *in vitro* experiments were performed under the guidance of the Animal Health Research Center (AHRC) in an approved biosafety level 3 laboratory with the use of a powered air-purifying respirator, according to “UGA Laboratory Safety Guidelines”. Influenza virus A/Anhui/1/2013 (A/Anhui; H7N9) and A/Philippines/2/82-X79 (A/Phi; H3N2) were propagated in embryonated eggs. Influenza A/California/04/09 (A/Ca; H1N1) was propagated in Madin-Darby canine kidney (MDCK) cells (ATCC CCL-34). Virus was titrated on MDCK cells to determine TCID_50_ values. The human type II pulmonary epithelial cell line A549 (ATCC CCL-185) was used for transfections and infections and cells were maintained in Dulbecco's Modified Eagle's Medium (DMEM: HyClone GELifeSciences) supplemented with 5% heat-inactivated fetal bovine serum (FBS) (HyClone, Logan, UT) in a 37°C incubator with 5% CO_2_. Virus propagation in embryonated eggs was carried out in strict accordance with the recommendations by the University of Georgia Institutional Animal Care and Use Committee (IACUC). The protocol was approved by the University of Georgia IACUC (A2013 06-016-Y3-A3 Title: NIAID Centers of Excellence for Influenza Research and Surveillance: Egg Usage).

### High-throughput miRNA inhibitor transfection screening

The miRNA inhibitor screen was performed in A549 cells as shown in Fig 1A. miRNA inhibitors (Dharmacon, GELifeSciences) were reverse-transfected into A549 cells at a final concentration of 20 nM in 0.3% DharmaFect1 (Dharmacon, GELifeSciences) transfection reagent. Non-targeting miRNA inhibitor controls (Dharmacon, GELifeSciences) were used in all assays as negative controls and siRNAs targeting NP (siNP) was used as positive controls. Briefly, using a 96-well format, miRNA inhibitors were mixed with DharmaFect1 reagent in serum-free medium (Opti-MEM, GELifeSciences) and incubated at room temperature for 20 min. A549 cells were then added at 10,000 cells/well in DMEM supplemented with 10% FBS using Liqudator96 (Mettler Toledo). 48 h post-transfection, the cells were infected with H7N9 in 100 μl of DMEM supplemented with 2% FBS, at a MOI 0.1. 24 h pi, the cells were fixed with methanol acetone (80:20) for 15 min and stored until analysis by ELISA.

### ELISA assay

The miRNA primary screen to identify host genes involved in down-regulating H7N9 virus replication in A549 cells was completed using a NP-specific ELISA assay. The fixed cells were incubated with a mouse anti-NP monoclonal antibody (ATCC H16-L10-4R5) at 37°C for 1 h and then incubated with an HRP-conjugated goat anti-mouse secondary (Invitrogen). Afterwards, the plates were washed with PBS followed by 15 min incubation with HRP-substrate solution (SureBlue TMB). H_2_SO_4_ stop solution was then added to the plates to terminate the reaction. The plates were scanned with a plate spectrophotometer at a wavelength of 450 nm. The OD values of experimental wells were normalized to the control wells and converted to a Z-score to create a hierarchical list containing miRNAs in the entire screen according to methods described previously [20]. In this study, all hits with a Z-score ≤ -1.5 and cytotoxicity (ToxiLight™ bioassay kit; Lonza) below 5% were subject to further validation in A549 cells.

### Validation assays

miRNA hairpin inhibitors and mimics and ON-TARGETplus siRNAs (Dharmacon, GE LifeSciences) were reverse-transfected with 0.3% DharmaFect1 transfection reagent into A549 cells at a final concentration of 25 nM and 50 nM, respectively. Non-targeting miRNA inhibitor controls (Dharmacon, GELifeSciences) were used as negative controls and siRNAs targeting NP (siNP) was used as positive controls. Briefly, using a 96-well format, miRNA inhibitors and mimics and siRNAs were mixed with DharmaFect1 transfection reagent in serum-free medium (HBSS, Invitrogen) and incubated at room temperature for 20 min. A549 cells were then added at 10,000 cells/well in DMEM supplemented with 5% FBS. 48 h post-transfection, the cells were infected with influenza A viruses while the transfection medium was tested for cytotoxicity with a ToxiLight™ bioassay kit (Lonza). miRNA inhibitors and mimics exceeding a toxicity of 5% were excluded for further validation.

48 h pi, the cell monolayers were fixed with 10% buffered formalin and permeabilized with Triton X-100. Cells were stained with a mouse anti-NP monoclonal antibody (ATCC H16-L10-4R5) and subsequently with a secondary Alexa Fluor 488 anti-mouse antibody (Life Technologies) each for 1h at room temperature. Cells were counterstained with DAPI to quantify the number of infected cells with Cellomics ArrayScan (Thermo Scientific). Supernatants were used to determine TCID_50_ titers on confluent MDCK cells. 10-fold serial dilutions were added to MDCK cells in a 96-well plate format and incubated for 72 h at 37°C and 5% CO_2_. Supernatants from MDCK cells were then tested for hemagglutination with 0.5% turkey red blood cells. TCID_50_ values were calculated using the Spearman Karber method [45, 46]. All infections were performed in the presence of 1 μg/mL (L-tosylamido-2-phenyl)ethyl chloro-methyl ketone treated (TPCK) trypsin (Worthington Biochemical) in modified Eagle’s medium (MEM) supplemented with 0.3% bovine serum albumin (Gibco, Life Technologies).

### RNA isolation and qRT-PCR

Total RNA was isolated using TRIzol (Invitrogen Life Technologies). The concentration of total isolated RNA was measured by using a Microplate Spectrophotometer (Epoch, BioTek). qRT-PCR was used to validate mRNA and miRNA expression changes using the Stratagene Mx3005P real-time PCR system (Agilent Technologies). The reverse transcription reactions were performed using a miRNA 1st-Strand cDNA Synthesis Kit (Agilent Technologies) using 500 ng total RNA for each reaction. qRT-PCR was performed using the Brilliant III Ultra-Fast SYBR® Green QRT-PCR Master Mix (Agilent Technologies) to determine miRNA and mRNA expression, and data were normalized to 18S expression using the 2^-ΔΔCt^ method. Primer sequence for miR-664 was, 5’-TATTCATTTATCCCCAGCCTACA-3’ (forward primer) and a universal reverse primer. Primers sequences for LIF were, 5’-ACAGAGCCTTTGCGTGAAAC-3’ (forward primer) and 5’-TGGTCCACACCAGCAGATAA-3’ (reverse primer). Primer sequences for NEK7 were, 5’-CACCTGTTCCTCAGTTCCAAC-3’ (forward primer) and 5’-CTCCATCCAAGAGACAGGCTG-3’ (reverse primer).

### Statistical analysis

All experiments were performed in biological triplicates and technical duplicates and have been repeated at least twice. Data are expressed as means ± SEM. Differences between different biological groups were compared by one-way analysis of variance (ANOVA). Individual differences between groups were tested by multiple comparison and analysis using the Tukey post-test. Pairs of groups were compared by Student’s t-test (two tailed). P values for significance were set at 0.05, unless otherwise stated. All analysis was performed using Graphpad Prism Software (Version 5 for Windows).

## Supporting Information

S1 Fig siRNA successfully knocks down expression of targeted genes. A549 cells were transfected with siRNA against LIF and NEK7 at 50 nM for 48 h. Cells were infected with A/Ca (H1N1) at MOI 0.1. RNA was extracted for gene expression analysis of A) LIF and B) NEK7 using RT-qPCR. Expression is normalized to 18S and relative to non-infected cells. Data are from 3 replicate wells ± SEM. *p<0.05, *** p<0.001.
(TIFF)
