# Supplementary figures and images for: MicroRNA Regulation of Human Genes Essential for Influenza A (H7N9) Replication
Source: PLoS One. 2016 May 11;11(5):e0155104. doi: 10.1371/journal.pone.0155104 (PMC4864377; doi:10.1371/journal.pone.0155104)

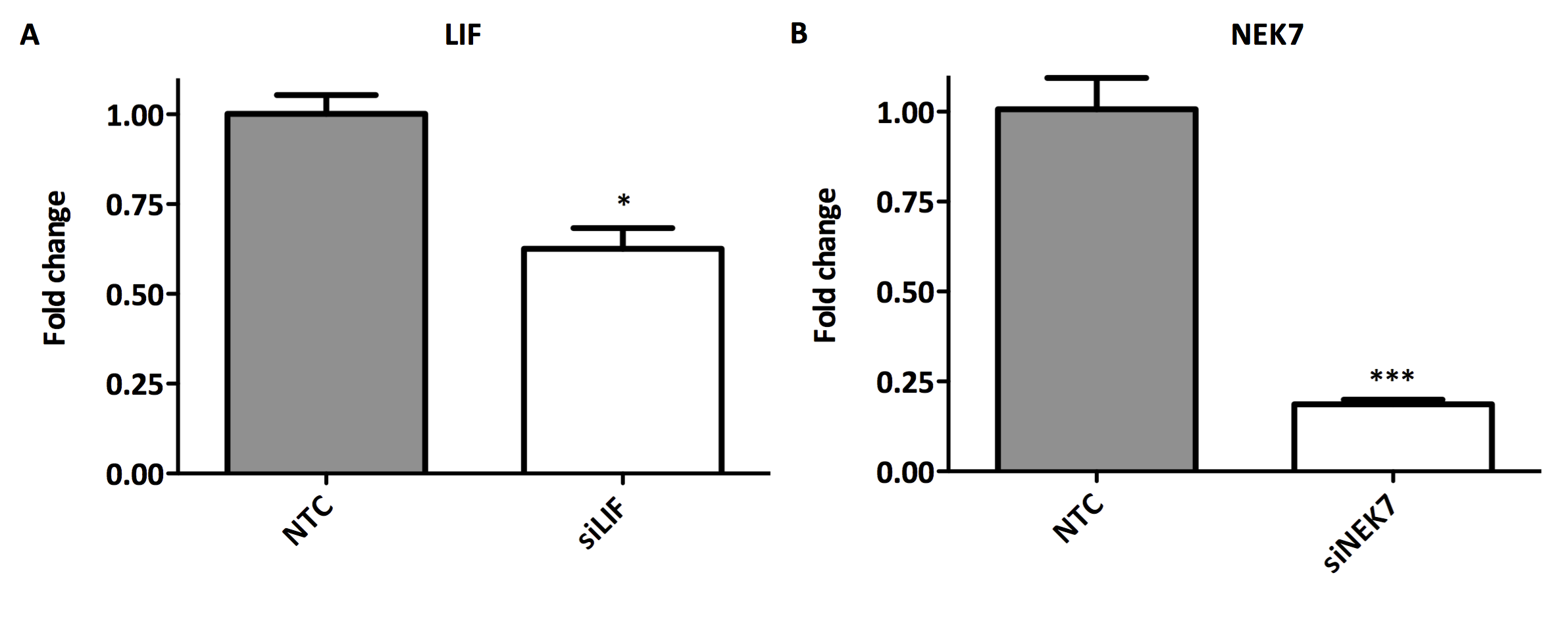

Supplement: S1 Fig — A549 cells were transfected with siRNA against LIF and NEK7 at 50 nM for 48 h. Cells were infected with A/Ca (H1N1) at MOI 0.1. RNA was extracted for gene expression analysis of A) LIF and B) NEK7 using RT-qPCR. Expression is normalized to 18S and relative to non-infected cells. Data are from 3 replicate wells ± SEM. *p<0.05, *** p<0.001. (TIFF) [file pone.0155104.s001.tiff]
